# Automatic Extraction of Optimal Endmembers from Airborne Hyperspectral Imagery Using Iterative Error Analysis (IEA) and Spectral Discrimination Measurements

**DOI:** 10.3390/s150202593

**Published:** 2015-01-23

**Authors:** Ahram Song, Anjin Chang, Jaewan Choi, Seokkeun Choi, Yongil Kim

**Affiliations:** 1 Department of Civil and Environmental Engineering, Seoul National University, 1, Gwanak-ro, Gwanak-gu, Seoul 151-742, Korea; E-Mail: yik@snu.ac.kr; 2 School of Earth and Environmental Sciences, Seoul National University, 1, Gwanak-ro, Gwanak-gu, Seoul 151-742, Korea; 3 School of Civil Engineering, Chungbuk National University, 1 Chungdae-ro, Seowon-gu, Cheongju, Chungbuk 361-763, Korea; E-Mails: jaewanchoi@chungbuk.ac.kr (J.C.); skchoi@chungbuk.ac.kr (S.C.)

**Keywords:** endmember extraction, optimal endmembers, hyperspectral, IEA

## Abstract

Pure surface materials denoted by endmembers play an important role in hyperspectral processing in various fields. Many endmember extraction algorithms (EEAs) have been proposed to find appropriate endmember sets. Most studies involving the automatic extraction of appropriate endmembers without *a priori* information have focused on N-FINDR. Although there are many different versions of N-FINDR algorithms, computational complexity issues still remain and these algorithms cannot consider the case where spectrally mixed materials are extracted as final endmembers. A sequential endmember extraction-based algorithm may be more effective when the number of endmembers to be extracted is unknown. In this study, we propose a simple but accurate method to automatically determine the optimal endmembers using such a method. The proposed method consists of three steps for determining the proper number of endmembers and for removing endmembers that are repeated or contain mixed signatures using the Root Mean Square Error (RMSE) images obtained from Iterative Error Analysis (IEA) and spectral discrimination measurements. A synthetic hyperpsectral image and two different airborne images such as Airborne Imaging Spectrometer for Application (AISA) and Compact Airborne Spectrographic Imager (CASI) data were tested using the proposed method, and our experimental results indicate that the final endmember set contained all of the distinct signatures without redundant endmembers and errors from mixed materials.

## 1. Introduction

Airborne hyperspectral data of high spatial and spectral resolutions contain many unknown signals. Identifying the number of distinct signatures that are present in a scene by visual analysis or *a priori* knowledge is often difficult, but doing so is important [1]. Pure surface materials denoted by endmembers need to be known for spectral mixture analysis, which is a popular technique for analysing hyperspectral remote sensing data [2]. Endmembers also play an important role in various fields, including classification [3–5], target or anomaly detection [6–8] and environmental monitoring and risk prevention and response [9–12].

Choosing a method of endmember extraction depends on the type of remote sensing data and the purpose of the data processing. One common approach is to use previously constructed spectral libraries, such as those from the Jet Propulsion Laboratory (JPL), Johns Hopkins University (JHU), and the United States Geological Survey (USGS) [13]. However, most existing spectral libraries include laboratory sources that were not acquired under the same conditions as one's collected data. An image endmember method that extracts pure endmember pixels from a scene is preferred in many hyperspectral processes (e.g., spectral unmixing analysis) because this approach increases the ease of accurately extracting endmembers and implementing the extracted endmembers [14]. Over the previous decade, several algorithms have been developed for the direct extraction of spectral endmembers from the hyperpsectral data. The algorithms can easily find the features in the hyperspectral scene and collect the same scale data such as the number of band [15]. The Pixel Purity Index (PPI) extracts endmember pixels by iterative processing based on projections of corresponding random dimensional vectors [16]. Neville *et al.* [17] proposed an endmember extraction algorithm based on an iterative unmixing process and error analysis. In addition, automatic processes that define the simplex based on the maximum volume, such as the N-FINDR algorithm, the Vertex Component Analysis (VCA) algorithm and the Successive Projection Algorithm (SPA), have been proposed [18–20]. Although these algorithms are limited by the assumption of the presence of pure signatures in a scene, these endmember extraction algorithms (EEAs) are widely used and developed due to their ease of computation and clear basis [21]. Many EEAs involve an iterative process, and it is therefore necessary to determine certain stopping rules based on an error threshold, ε, or the desired number of endmembers, *p*. Because ε depends on the properties of the data, it is difficult to pre-determine the threshold without prior analysis of the data in many cases [22]. Therefore, it is essential to determine an appropriate value of *p* for terminating the algorithm. However, no corresponding criteria have been established for many EEAs, and this issue remains unresolved [23]. If *p* is set to higher value than the number of pure signatures in a given dataset, then mixed or interfering substances may be extracted; conversely, if *p* is set to too low value, then the EEAs may not extract all of the pure pixels as endmembers [22].

Various concepts for setting an appropriate value of *p* have been proposed. Most studies of determining the appropriate value of *p* have focused on N-FINDR; thus, there are many versions of N-FINDR algorithms. Plaza and Chang [24] modified the N-FINDR algorithm using an initialisation of the endmembers and using Virtual Dimensionality (VD) to determine how many endmembers need to be generated by N-FINDR. VD identifies distinct signatures in the hyperspectral data and can identify not only pure signatures but also anomalies without *a priori* knowledge [24,25]. VD is capable of determining the appropriate number of distinct signatures; however, the calculation is complex because the correlation eigenvalues and covariance eigenvalues of each spectral band must be determined and VD does not effectively work with hyperspectral images [26]. Chang *et al.*, [27] proposed the random N-FINDR (RN-FINDR) in order to determine *p* automatically and resolve inconsistent final endmember selection problem. RN-FINDR selects intersection set through a random process which conducts two consecutive runs of original N-FINDR using the different initial endmember sets, and the method found commonly extracted endmembers form the different random initial endmember sets and decided them as final endmembers. However, there were possibilities that spectrally mixed or interfering substances could be selected as final endmembers in this method if those substances were extracted as repeated endmembers from the different initial endmember sets. Also, although RN-FINDR could determine the number of final endmembers automatically, it was recommended to use the VD estimates to avoid random guess of *p,* exhaustive search. One major problem of N-FINDR is computational complexity [28] and in order to reduce computation cost, a sequential endmember search method was implemented [29]. Du also showed that a sequential endmember extraction-based algorithm can improve the accuracy of extracted endmembers without a computational complexity of determining initial condition [2].

In this paper, we propose a new optimal endmember determination technique based on sequential endmember extraction to consider the spectral similarities of extracted endmembers and reduce the computational burden. The proposed method consists of three steps and determines the appropriate number of endmembers automatically, and this process removes impure and repeated endmembers using the total Root Mean Square Error (RMSE) generated from the Iterative Error Analysis (IEA) and spectral discrimination measurements. In order to show the efficiency of our method, we performed a comparative performance analysis of the proposed method and RN-FINDR using a synthetic image and two different airborne hyperspectral images.

## 2. The Proposed Method of Optimal Endmember Extraction

### 2.1. Linear Mixture Model in the Hyperspectral Image

One of the most popular and important methods for analysing hyperspectral data is spectral unmixing. A few spectral signatures jointly occupy a single pixel when the spatial resolution of the hyperspectral image is too coarse to distinguish between different materials on the ground. The measured spectral signature is thus a composite of the individual spectra. Although sub-pixel nonlinear mixing can be important in certain types of analyses, the effects of multiple scattering in the majority of applications are assumed to be negligible when a linear model is used [15,30]. The key task when using a linear mixture model is to find an appropriate set of pure endmembers (spectral signatures). Therefore, it is very important to extract accurate and optimum endmembers for the unmixing and for analysis of the hyperspectral data [31,32].

Let r = (*r*_1_, *r*_2_,…,*r_n_*)*^T^* be a pixel in the hyperspectral image composed of *n* spectral bands and *E* = [*E*_1_,*E*_2_,…,*E_p_*] be the spectrally pure constituent endmembers of the *p* materials, where *E_i_* = [*E_i_*_1_,*E_i_*_2_,…,*E_in_*]*^T^* means the *i-*th endmember that has spectral reflectance *E_in_* corresponding to the *n-*th bands. To define the linear mixture model in mathematical terms, it is assumed that each acquired pixel *r* can be represented as the linear combination of endmembers *E* weighted by an abundance vector *a* = (*a*_1_,*a*_2_,…, *a_p_*)*^T^* that represents the proportion of each endmember in the pixel under inspection, as follows:

(1) r=Ea+ɛ

where ε represents a source of additive noise (e.g., perturbation and modelling errors) [31]. In a given pixel, the fractional abundance *a_i_* represents the fractional area occupied by endmember *E_i_*. If it is assumed that the optimum endmembers are known, then the fractional abundance can be determined using the least-square approach [32]. Generally, in Equation (1), an abundance vector corresponding to pixel *r* can be calculated, as in Equation (2):

(2) $$a={\left(E^{T}E\right)}^{-1}E^{T}r$$

The fractional abundances are subject to two constraints: the abundance non-negativity constraint (ANC) and the abundance sum-to-one constraint (ASC). These two constraints are given, respectively [33], by these relationships:

(3) $$\text{Non}-\text{negativity}\;a_{i}\geq 0,i=1,\ldots ,p\text{and}$$

(4) $$\text{Sum}-\text{to}-\text{one}\sum_{i=1}^{p}a_{i}=1$$

In addition, the ANC problem can be solved using various methods, including constraint least-square-based optimisation techniques and projection-based algorithms [34,35].

### 2.2. IEA for Initial Endmember Set Extraction

**Figure 1.**
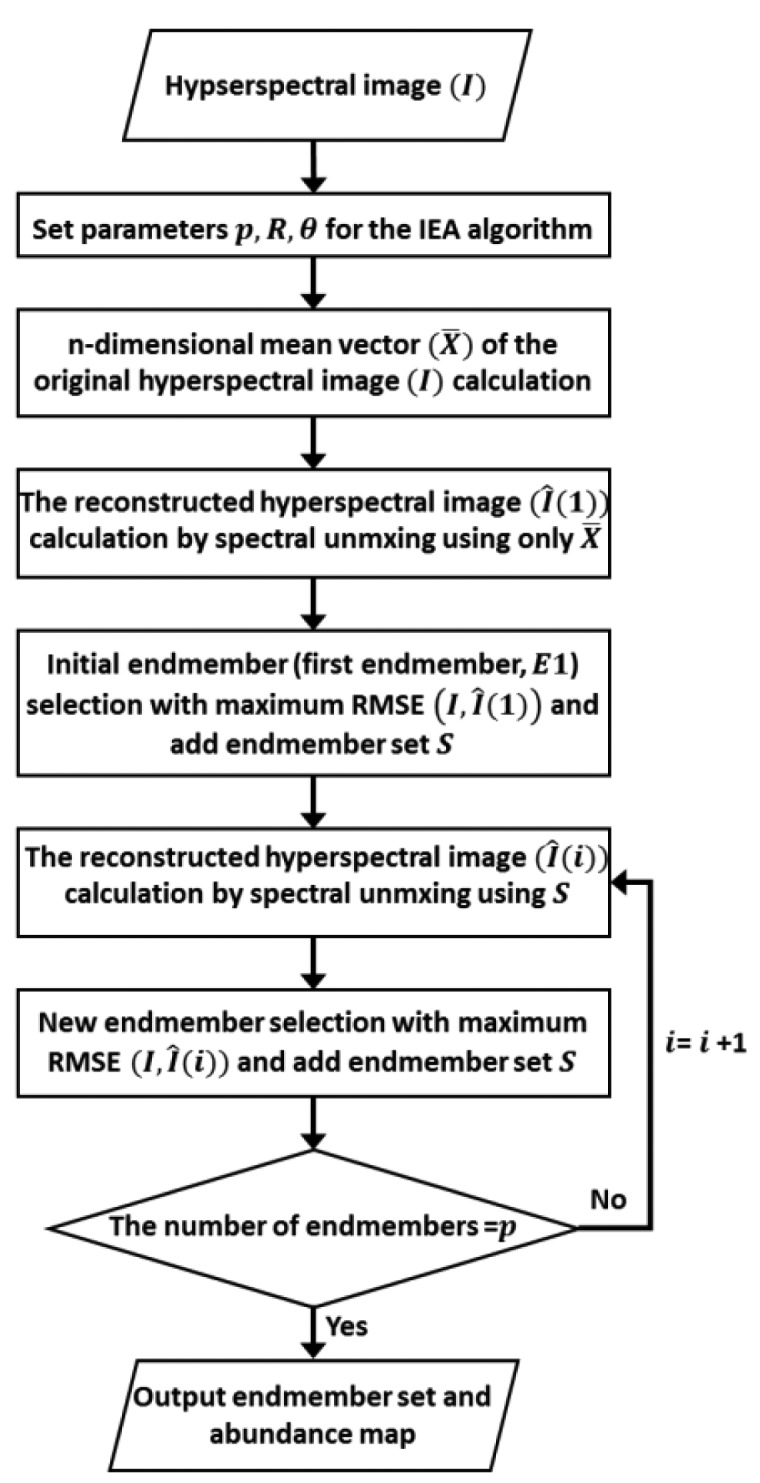
Logical flow of the IEA algorithm.

The IEA is one of the popular, sequential, linear constrained endmember extraction algorithms based on the linear mixture model. The algorithm identifies endmembers one by one based on previously extracted endmembers. The pixels, which minimise the remaining error in the unmixed image, are selected as endmembers each time. The IEA method involves a series of linear constrained spectral unmixing steps to search for endmembers that minimise the remaining error in an abundance map [32,33]. Figure 1 shows the logic flow of the IEA algorithm.

Three input parameters should be determined in advance: *p*, *R*, and θ. The parameter *p* is the desired number of endmembers. *R* is the number of pixels with the largest number of errors, selected from the error image, and θ is the spectral angle (SA) between spectral vectors [15]. The SA is widely used in remote sensing to calculate the angle between two spectra and is defined as:

(5) $$\text{SA}\left({\text{s}}_{i},{\text{s}}_{\text{j}}\right)=co{\text{s}}^{-1}\left(\frac{\langle s_{i},s_{\text{j}}\rangle }{\left\|s_{i}\right\|\left\|s_{\text{j}}\right\|}\right)$$

where 〈***s****_i_*, ***s****_j_* 〉 is the inner product between the spectral signatures *s_i_* and *s_j_* and ‖***s***‖ is the vector magnitude [36,37]. First, the IEA algorithm is used to calculate the mean of the original image (*X̄*), and this mean is used as the initial vector. Using this initial vector, constrained linear spectral unmixing is performed on the original hyperspectral image (*I*), and an abundance value (*i.e.*, the process output) is multiplied by the initial vector to produce the reconstructed image (*Î*(1)). A RMSE(*I*, *Î*(1)) can be defined as the average of the RMSEs between *I* and *Î*(1). When it is assumed that the original image is composed of *n* bands and *I_k_*(*i*,*j*) denotes the pixel value at *k*-th bands with spatial coordinates (*i*,*j*), then the RMSE between the original and the reconstructed image can be calculated using the following expression:

(6) $$\text{RMSE}\left(\text{I},\hat{I}\right)=\left(\frac{1}{w\times c}\right)\sum_{i=1}^{w}\sum_{j=1}^{c}{\left(\frac{1}{n}\sum_{k=1}^{n}{\left[I_{k}\left(i,j\right)-{\hat{I}}_{k}\left(i,j\right)\right]}^{2}\right)}^{\frac{1}{2}}$$

where *w* denotes the number of rows and *c* denotes the number of columns in the original image (*I*). A subset of *R* consisting of pixels within an angle θ from the maximum error vector is calculated, and these pixels are averaged to generate the new endmember vector. The first endmember, ***E*_1_** (*i* = 1) of endmember set S, is the mean vector of the largest error pixels in *R* that are within spectral angle θ and farthest from the initial vector. Using the *i*-th endmember (*i* = 1, 2, …*,p*), the constrained linear spectral unmixing is performed again to determine the (*i* + 1)-th endmember from the (*i* + 1)-th RMSE image. The (*i* + 1)-th endmember is the mean pixel in *R*(θ)among the pixels in *(i* + 1)-th RMSE image that have the largest errors. This process is repeated using the *(i* + 1*)*-th endmembers until the stopping rule is satisfied; the stopping rule is to obtain a certain predetermined number of endmembers or to reach a predetermined error tolerance [33]. In other words, the algorithm is terminated when the number of extracted endmembers equals *p* or the unmixing error values decrease below the threshold.

### 2.3. Determination of Optimal Endmembers

**Figure 2.**
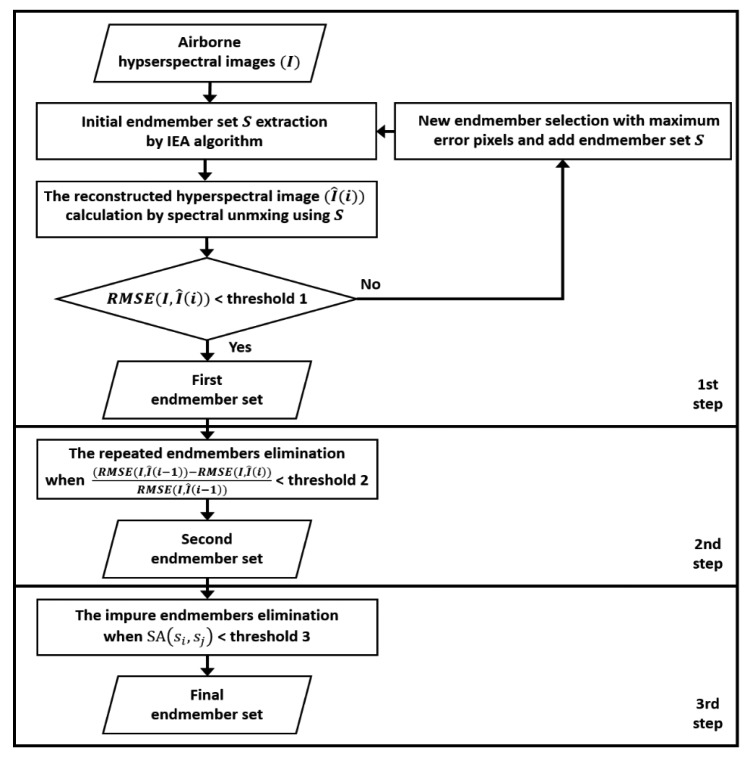
Flow chart of the proposed method.

The objectives of this study are focused on automatic determination of *p* and removal of impure and mixed endmembers from initial endmember set. The optimal endmembers were identified using the following three steps: (1) extracting an initial set of endmembers using the RMSE generated from the IEA; (2) eliminating repeated endmembers; and (3) separating impure endmembers that do not correspond to a single material but instead consist of two or more signatures. Figure 2 shows the flow chart of the proposed method for determination the optimal endmembers.

We did not predetermine the desired number of endmembers. Instead, the IEA algorithm was continuously applied until the RMSE was approximately equal to zero. Although the two constraint conditions of the linear mixture model were generally not satisfied in the case of real hyperspectral data, RMSE values of all pixels generated via constrained linear spectral unmixing (with the abundance non-negativity constraint and abundance sum-to-one constraint) are almost close to zero when all of the pure signatures are adequately extracted from a given data set.

The RMSE continuously decreases with the identification of new endmembers by the IEA algorithm. For example, the RMSE (*I*, *Î*(i)) generated from the *i*-th endmember set [***E*_1_**,***E*_2_**, …, ***E****_i_*] is lower than RMSE (*I*, *Î*(*i* − 1)) generated from [***E*_1_**,***E*_2_**, …, ***E****_i_***_−1_**] because *E_i_* explains the new component of a pixel that cannot be described with the (*i −* 1)-th endmember set. However, IEA had the possibility of extracting repeated materials as a new endmember enven though pure materials still were remained in the scene, due to noise and interfering signatures in specific bands. If *i*-th endmember were very similar materials with already extracted endmembers, there were little difference between RMSE (*I*, *Î*(*i* − 1)) and RMSE (*I*, *Î*(*i*)). In other words, if RMSE (*I*, *Î*(*i*)) and RMSE (*I*, *Î*(*I −* 1)) have similar values, then *E_i_* is assumed to be a same material with previously extracted endmembers. Therefore, in this study, if the rate of change between RMSE (*I*, *Î*(*i*))and RMSE (*I*, *Î*(*I −* 1)) is negative and it is less than the threshold, then *E_i_* is considered as a repeated endmember and was removed from the first set of endmembers.

Although the rate of decrease may fall below the threshold, all endmembers do not necessarily represent pure signatures. If the set of endmembers (excluding repeated endmembers) is larger than the true number of pure materials in the scene, then certain endmembers might be mixed or redundant signatures. The RMSE might decrease significantly with the presence of numerous mixed pixels, which might be represented by certain endmembers. To separate impure signatures from the endmember set after the second step and to extract the final optimum set of endmembers, we used a spectral discrimination measure. We assumed that if certain endmembers had a small spectral angle approximately equal to those of two or more previously extracted endmembers, then these putative endmembers would represent mixed signatures. Most sequential EEAs find different endmember types earlier [22]. Therefore, we confined that initial three endmembers represented pure materials to apply the assumption.

As mentioned earlier, the spectral angle was used as the spectral discrimination measure. A smaller SA corresponds to a higher similarity. When the *j*-th (*j* > 3) endmember had an SA similar to two or more previously extracted endmembers, then the endmember was considered to be a mixed endmember and was removed. The threshold for estimating similarity is selected from the SA values of initial three endmembers. Using the student's t-distribution, we could estimate confidence interval range of pure material's mean SA. Student's t distribution estimates an interval of true mean (*μ*) from the sample data with significance level (*α*) and degrees of freedom (*m*−1), as follows [38]:

(7) $$\bar{X}-t_{\left(\frac{\alpha }{2},m-1\right)}\frac{s}{\sqrt{m}}\leq \mu \leq \bar{X}+t_{\left(\frac{\alpha }{2},m-1\right)}\frac{s}{\sqrt{m}}$$

*X̄* is the sample mean, *s* is the sample standard deviation and *m* is the number of sample data. In this study, the minimum value from the estimated mean range of SA was the threshold which determined the similarity about the whole scene and it is dependent on material's types located on a scene.

In the proposed method, we were able to automatically determine the optimum number of endmembers (*p*) and the optimum endmember set, excluding mixed and impure signatures and including only pure signatures.

## 3. Study Area and Data

### 3.1. Synthetic Hyperspectral Image

**Figure 3.**
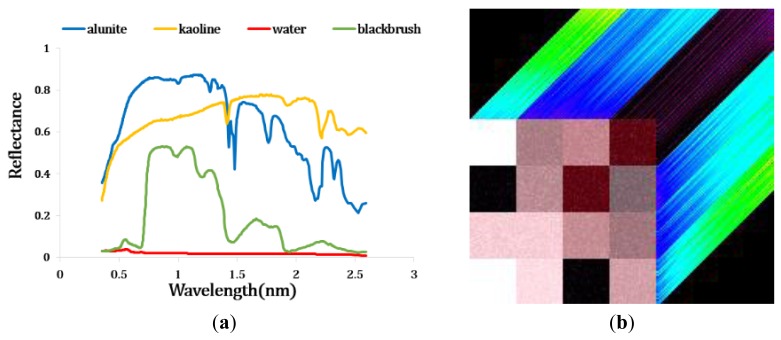
(**a**) Spectra of four materials obtained from USGS spectral library. (**b**) Synthetic hyperspectral image with SNR 30 dB.

In order to evaluate the efficiency of the proposed method and verify our threshold values, a synthetic hyperspectral image was used. The synthetic image had a size of 120 × 120 pixels and 437 bands with wavelength of 351∼2592 nm. It consisted of four reflectance signatures such as water, two mineral (alunite, kaolinite), and one vegetation (blackbrush leaves) obtained from the USGS digital library (Figure 3a). To make the scene more realistic, the white Gaussian noise was added and SNR was set to 30 dB [39]. The synthetic image contains twelve region, and each area had different endmembers and abundance values (Figure 3b) [14].

### 3.2. Real Hyperspectral Image

**Figure 4.**
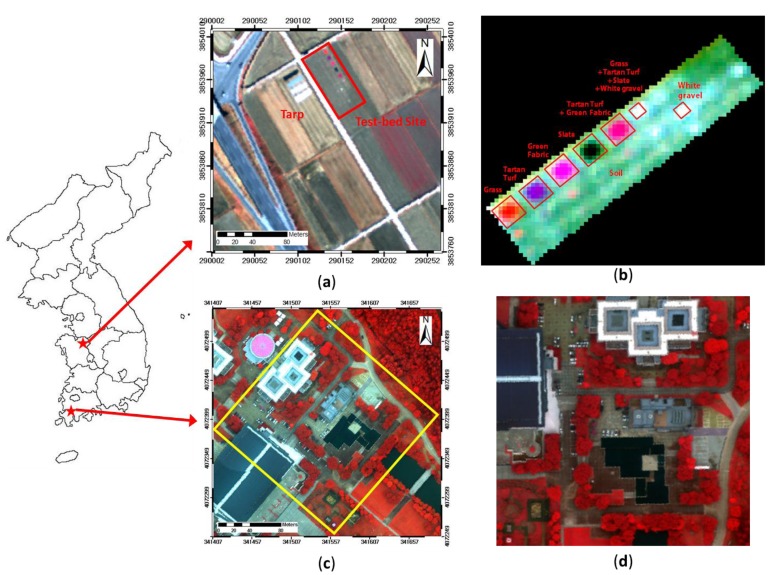
(**a**) AISA image featuring the test bed in the UTM (WGS 84, 52S zone) coordinate system: red is band 97 (845–849 nm), green is band 65 (688–693 nm), and blue is band 33 (539–544 nm); (**b**) Non-geometrically calibrated AISA subset image; (**c**) CASI image in the UTM (WGS 84, 52S zone) coordinate system: red is band 27 (809–847 nm), green is band 16 (599–618 nm), and blue is band 10 (484–504 nm); (**d**) Non-geometrically calibrated CASI subset image.

The proposed method also was applied in real hyeprsepctral data collected from the Airborne Imaging Spectroradiometer for Application (AISA) and the Compact Airborne Spectrographic Imager (CASI) datasets. The AISA data were acquired on 1 December 2012 in Yeongam, South Korea (Longitude 126°42′21.5″, Latitude 33°48′23″) (Figure 4a). The spatial resolution of the AISA data was 1 m, and the data contained 128 bands in the spectral range of 400 nm to 970 nm. A test bed was constructed in a scene covered by the AISA data. This test bed consisted of plots of various sizes containing various substances, such as grass, tartan turf, green fabric, slate, white gravel, and native soil. The test bed included four plots of pure material and one plot of mixed material, each measuring 4 m × 4 m, and one plot of pure white gravel and one plot of mixed material, each measuring 2 m × 2 m; thus, two plots of mixed materials were included (Figure 4b). A subset image of the test bed was used to enable the use of reference data.

The CASI airborne hyperspectral image was acquired on 26 October 2010 in Cheonan, South Korea (Longitude 127°13′30″, Latitude 36°47′5″) (Figure 4c). The spatial resolution of the CASI data was 0.5 m, and the data contained 36 bands in the spectral range of 365 nm to 1050 nm. The scene contained artificial materials such as roads and buildings and natural materials such as water and various types of vegetation (Figure 4d).

The AISA and CASI hyperspectral data were calibrated using the vicarious radiometric calibration method for conversion into the spectral reflectance values because the raw data were digital numbers (DNs) or the radiance of the sensors. The empirical line calibration (ELM) method was adopted based on the field data measured using a spectroradiometer. The linear relational equation was evaluated by comparing the radiance value in the scene with the ground spectral reflectance value [40]. Each pixel in the hyperspectral image was converted using the linear equation. The AISA data included 4 types of tarps (3.5%, 23%, 35%, and 53%), and the CASI data included eight types of materials on the ground. In addition, the hyperspectral AISA and CASI data used in this study were not calibrated geometrically because errors in the spectral data may be caused by interpolation, which is one of several types of processing errors in the geometric calibration.

## 4. Results and Discussion

### 4.1. Synthetic Image Experiment

The proposed method extracted optimal endmembers from the synthetic hyperspectral image. We set the IEA algorithm parameters to values of *R* = 1and θ= 0 to extract a representative pixel and unchanged value of a real target in the hyperspectral data and to avoid spectral averaging to increase the spectral purity of the obtained endmembers [33].

**Figure 5.**
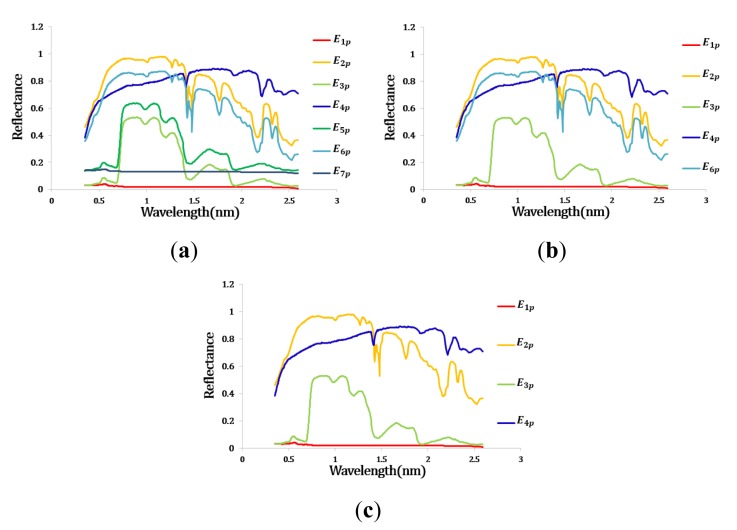
The spectra of the endmember set for the synthetic hyperspectral image. (**a**) After the first step; (**b**) After the second step; (**c**) After the final steps.

Table 1 showed the RMSE and rate of change of extracted endmembers. The RMSE decreased with new endmembers and gathered almost zero. The RMSE did not reach zero because the real data contained noise. Therefore, IEA continuously extracted endmembers until the RMSE (*I*,*Î*(i)) was less than under 1.0 × 10^−2^ (first threshold). After first steps, first endmember set contained seven endmembers (Figure 5a).

The endmembers having same spectral properties with previous endmembers had significantly small rate of decrease. If the rate of decrease were less than 0.1 (second threshold), the proposed method eliminated the endmembers as repeated materials. In this case, *E*_5_*_p_* (5-th endmember obtained using the proposed algorithm) and *E*_7_*_p_* were eliminated as repeated endmembers (Figure 5b).

After removing repeated endmembers, SAs were calculated between remained endmembers in order to separate the mixed endmembers (Table 2). The mean confidence interval range was generated from *t-*distribution with confidence level 80% and degree of freedom 2. The mean and standard deviation of initial endmembers's SA were 0.632 and 0.2743, respectively. The minimum value from the estimated mean range of SA was 0.334 (third threshold). The endmembers having SA less than threshold 3 such as *E*_6_*_p_* were eliminated as a mixed signatures.

After the whole process, final endmember set contained four endmembers and the spectra of final endmembers represented initial four spectral signatures of pure materials (water, alunite, kaolinite, blackbrush leaves) (Figure 5c).

### 4.2. Real Image Experiment

Optimal endmembers were extracted from the two airborne hyperspectral datasets. To compare the effectiveness of the proposed method, we compared our results with those from RN-FINDR, which determines final endmembers without the prior information of *p* and resolves inconsistent selection of final endmembers from randomly selected initial endmembers [27].

One simple method of evaluation has been to compare the endmembers with available ground-truth spectra. The quality of endmember set can be evaluated by analysing the spatial distribution of abundance map [15]. Abundance map can represent relative fraction of the endmembers as well as spatial distribution. These abundances are estimated using a fully constrained linear spectral unmixing approach, and they are usually used to assess the distribution of each material, which allows for evaluation of the endmembers from a spatial viewpoint. Therefore the quality of a suite of endmembers can be evaluated by looking at the spatial distribution of abundance map.

**Figure 6.**
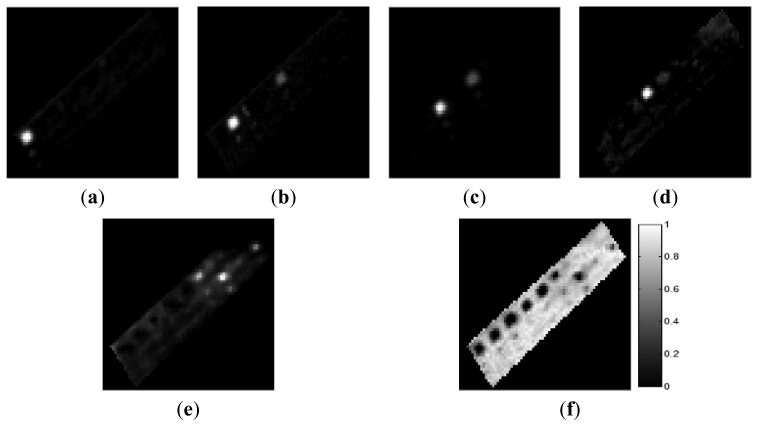
Abundance maps of ground-truth materials in the AISA data. (**a**) Grass; (**b**) Tartan turf; (**c**) Green fabric; (**d**) Slate; (**e**) White gravel; (**f**) Soil.

**Figure 7.**
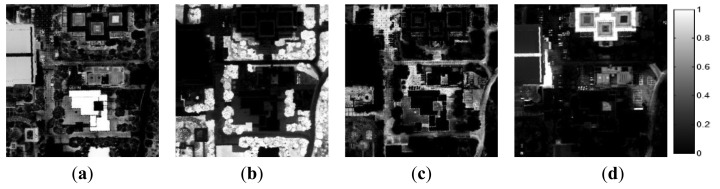
Abundance maps of ground-truth materials in the CASI data. (**a**) Water; (**b**) Vegetation; (**c**) Roads; (**d**) Buildings.

The ground-truth data were obtained from the scenes by visual analysis based on prior information regarding the scenes. In the AISA data, we defined the six materials comprising the test bed: grass, tartan turf, green fabric, slate, white gravel, and native soil. Abundance maps of the six ground-truth materials are shown in Figure 6. In the CASI data set, we defined four components comprising the ground-truth data: water, roads, buildings, and vegetation. Figure 7 shows the abundances of these four ground-truth materials. Each abundance map is the result of the specific properties of a certain material. The tile roof in Figure 7a exhibited relatively high values of the water component because this dark-coloured roof had spectral properties similar to those of water.

To evaluate the accuracy of the extracted endmembers, they were matched to the available ground-truth signatures based on similarities in their spectral values. In this case, we used SAs between those of the endmember and the ground-truth data. Then, we calculated the RMSE between the abundance maps. The use of these RMSE values is a simple method of evaluating the similarities between reference values and estimated values.

#### Optimal Endmember Extraction

**Figure 8.**
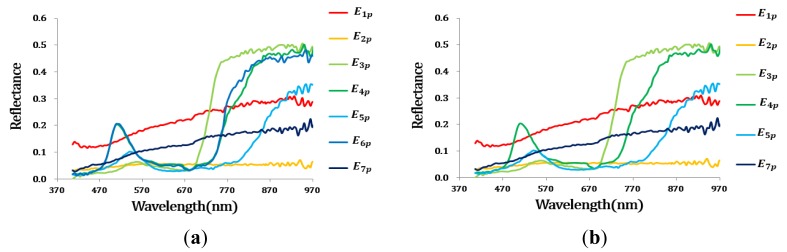
The spectra of the endmember set for the AISA data. (**a**) After the first step; (**b**) After the final step.

Initially, seven endmembers were extracted from the AISA data. The spectra of these initial endmembers are shown in Figure 8a. Among the initial endmember set, those endmembers for which the change from the previous endmember was small (≤0.1), such as *E*_6_*_p_* was eliminated as repeated endmembers (Table 3). Actually, *E*_4_*_p_* and *E*_6_*_p_* had very similar spectral signatures (Figure 8a). The minimum value from the estimated mean range of SA was 0.213. Since there were no endmembers which had a small SA to those of two or more previously extracted endmember, any endmember did not be removed in the third process (Table 4).

**Figure 9.**
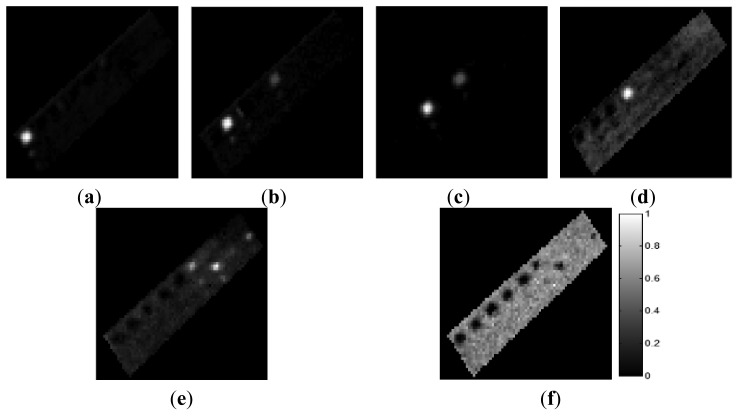
Abundance maps of the final endmembers in the AISA dataset obtained using the proposed method. (**a**) *E*_3_*_p_*, (**b**) *E*_5_*_p_*, (**c**) *E*_4_*_p_*, (**d**) *E*_2_*_p_*, (**e**) *E*_1_*_p_*, and (**f**) *E*_7_*_p_*.

The final endmember set contained six materials (Figure 8b), and the abundance maps of the final endmembers are shown in Figure 9. All six materials comprising the test bed were effectively extracted. Each endmember was matched with a specific ground-truth material based on their similar values. Although ***E*_1_***_p_* and ***E*_2_***_p_* corresponded to, respectively, the white gravel and slate in the ground-truth data, these endmembers also displayed relatively high abundance values in the area of native soil (Figures 9e and d). Because of the small size of the white gravel plot (2 × 2 m), the spectral pattern of this plot was affected by the surrounding soil. In addition, ***E*_1_***_p_* had a spectral pattern similar to that of the soil (Figure 8b). The slate's spectral properties were similar to those of the soil in terms of its low brightness. Therefore, the abundance values of ***E*_1_***_p_* and ***E*_2_***_p_* were relatively high in the area of soil. ***E*_3_***_p_*, ***E*_4_***_p_***,** and ***E*_5_***_p_* were matched with the grass, green fabric and tartan turf of the ground-truth data. ***E*_4_***_p_* and ***E*_5_***_p_* displayed high abundance values in the same areas. The corresponding areas were composed of tartan turf and green fabric in the same proportions. Therefore, these two endmembers exhibited high abundance values. ***E*_7_***_p_* was matched with the soil and represented the background of the test bed.

RN-FINDR used VD estimates as an initial value of *p* to avoid exhaustive process. And then, final endmembers were determined through automatic process, final endmembers obtained from RN-FINDR were different according to the threshold which determines whether the endmembers at different runs belong to same class or not, and the threshold value was defined empirically [27].

**Figure 10.**
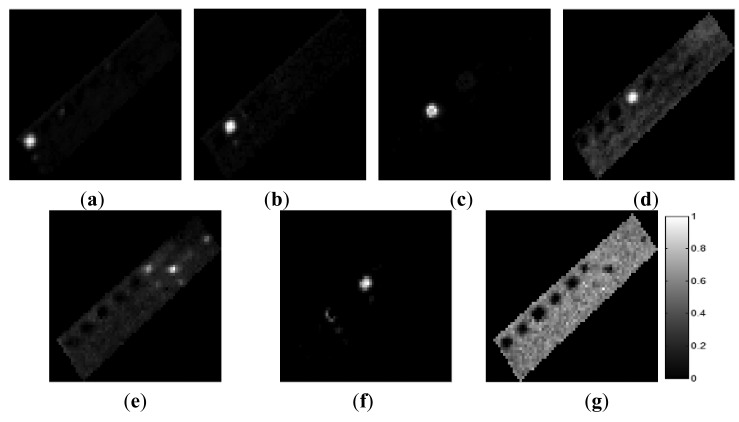
Abundance maps of the final endmembers in the AISA dataset obtained using RN-FINDR. (**a**) *E*_6_*_rn_*, (**b**) *E*_5_*_rn_*, (**c**) *E*_4_*_rn_*, (**d**) *E*_3_*_rn_*, (**e**) *E*_1_*_rn_*, (**f**) *E*_2_*_rn_* and (**g**) *E*_7_*_rn_*.

The final six endmembers, which were selected by RN-FINDR, contained a mixed substance instead of pure one. In order to extract all of pure materials and compare to identical with ground-truth, the threshold was reset to higher value than previous process and the seven endmembers were extracted as final endmember set. Figure 10 shows the abundance maps of the seven endmembers using the RN-FINDR algorithm. The seven endmembers mainly represent grass (***E*_6_***_rn_*), tartan turf (***E*_5_***_rn_*), green fabric (***E*_4_***_rn_*), slate (***E*_3_***_rn_*), white gravel (***E*_1_***_rn_*), mixed material (***E*_2_***_rn_*) and soil (***E*_7_***_rn_*). ***E*_2_***_rn_* was composed of tartan turf and green fabric. Since ***E*_2_***_rn_* was extracted by RN-FINDR, mixed area consisting of tartan turf and green fabric could not be explained by the components of ***E*_5_***_rn_* and ***E*_4_***_rn_* (Figure 10b,c).

To check the similarities between these results and the ground-truth data, the RMSEs between the abundance maps obtained using the two algorithms and the ground-truth data were calculated (Table 5). The endmembers extracted using the proposed method had lower RMSEs. Therefore, we conclude that our methodology efficiently determined the number and signatures of optimal endmembers.

**Figure 11.**
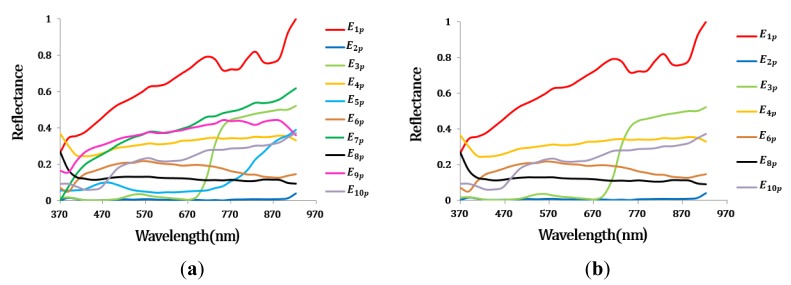

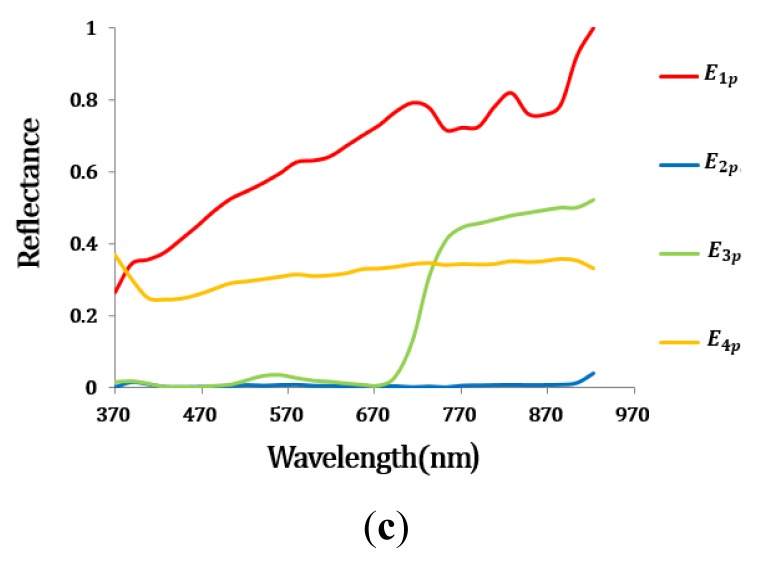
The spectra of the endmember sets for the CASI data. (**a**) After the first step; (**b**) After the second step; (**c**) After the final steps.

An additional CASI image was also tested using the proposed method. The initial endmember set contained ten materials. Repeated endmembers (*i.e.*,*E*_5_*_p_* , *E*_7_*_p_*, *E*_9_*_p_*) and impure endmembers (*i.e.*, *E*_6_*_p_*, *E*_8_*_p_*, *E*_10_*_p_*) were effectively eliminated within three steps (Tables 6 and 7). The threshold value of first and second step were equal to those of AISA, while the threshold of SA value in third step was 0.693. The spectra of the endmembers at each step are shown in Figure 11. After the three steps, six endmembers were eliminated, and the final four endmembers corresponded to buildings, vegetation, roads, and water.

**Figure 12.**
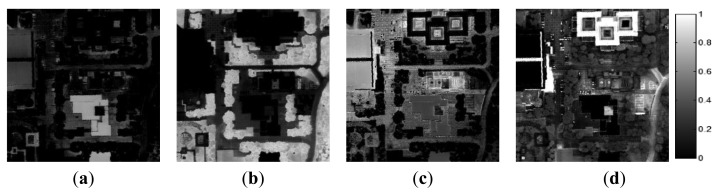
Abundance maps of the final endmembers in the CASI data obtained using the proposed method. (**a**) ***E*_2_***_p_*, (**b**) ***E*_3_***_p_*, (**c**) ***E*_4_***_p_*, and (**d**) ***E*_1_***_p_*.

Figure 12 shows the abundance values of the final endmembers obtained using the proposed method. ***E*_1_***_p_* was matched with the building component of the ground-truth data and corresponded to buildings and white paint (e.g., car park markings) (Figure 12d). This endmember corresponded to high-reflectance objects, and thus concrete road pavements also displayed high abundance values of this endmember.

**Figure 13.**
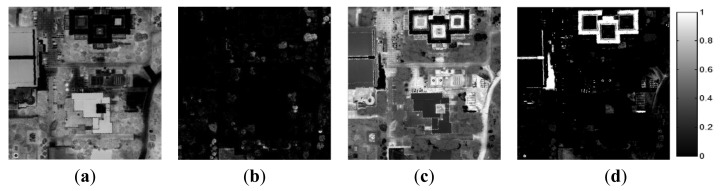
Abundance maps of the final endmembers in the CASI data obtained using RN-FINDR. (**a**) ***E*_1_***_rn_*, (**b**) ***E*_3_***_rn_*, (**c**) ***E*_4_***_rn_*, and (**d**) ***E*_2_***_rn_*.

***E*_2_***_p_* was matched with water in the ground-truth data and indicated water bodies in the scene. The step-sided square area in the middle of Figure 12a represents fountains, and the area below the fountains in the image represents small ponds. Because ***E*_2_***_p_* corresponded to dark objects, certain types of pavements and the low-reflectance tile roof also displayed relatively high abundance values of this endmember. ***E*_3_***_p_* corresponded to vegetation (Figure 12b). Finally, ***E*_4_***_p_* was matched with the roadway component of the ground-truth data and primarily corresponded to roadways and roofs with low reflectance values. ***E*_4_***_p_* corresponded to brighter objects than did ***E*_2_***_p_* (Figure 12c). Figure 13 shows the abundance maps obtained using the RN-FINDR algorithm. The abundance maps of RN-FINDR were only matched with few components comparing to those of the proposed method although there were various artificial materials such as roof, road, and pavement in the hyperspectral image. It implies that the proposed method could extract more accurate and purer endmembers. Based on the visual analysis, the endmembers obtained using the proposed method were represented in the abundance maps to a similar degree as in the ground-truth data and produced lower RMSEs than did those obtained using the RN-FINDR algorithm (Table 8).

Three endmembers among the four materials had lower RMSEs. The endmember obtained using RN-FINDR and matched with the building component in the ground-truth data, ***E*_2_***_rn_****,*** produced lower RMSE than did that obtained using the proposed method. The abundance map of ***E*_2_***_rn_* on Figure 13d showed high values on the boundaries of buildings and ground surfaces besides the roofs. ***E*_1_***_rn_* and ***E*_4_***_rn_* were matched with water and roadway components, respectively. They also displayed relatively high abundance values in the vegetation area (Figure 13a,c). On the other hand, ***E*_3_***_rn_* could explain only some portion of vegetation area with high reflectance although it was matched with vegetation component.

## 5. Conclusions

This paper presents a simple but accurate method of extracting optimal endmembers without prior knowledge the number of endmembers by adding two steps of processing after extracting endmember by IEA. First, we determined the number of initial endmember, *p*, automatically and extracted an initial set of endmembers using the RMSEs generated from the IEA. Then, we eliminated repeated endmembers and finally separated mixed endmembers that correspond not to single materials but consist of two or more signatures.

To demonstrate the efficiency of our methodology, the experiments were conducted using a synthetic data and two types of airborne hyperspectral images. The parameters were determined by using the synthetic data generated from USGS's spectral library and statistical method. The optimal endmembers were extracted from two airborne datasets with the reasonable threshold. The experimental results indicated that impure and repeated endmembers were effectively eliminated by the proposed method.

We compared the results of our proposed method with those of RN-FINDR algorithm, which is the improved version of N-FINDR algorithm and determines endmembers without the prior information of *p* value and inconsistent final endmember set. The accuracy was assessed using the RMSEs between the estimated abundance maps and ground-truth fractional maps. Generally, our method produced lower RMSEs than RN-FINDR, which indicates that the proposed method effectively extracts optimal endmembers from hyperspectral data.

## Figures and Tables

**Table 1.** RMSEs and rates of change of extracted endmembers. The values shown in bold italics are those of endmembers for which the rate of decrease is less than 0.1.

**Endmember**	**RMSE (**RMSE (*I*,*Î*(*i*))	**Rate of Decrease** $\left(\frac{\left(RMSE\right)\left(I,\hat{I}\left(i-1\right)\right)-RMSE\left(I,\hat{I}\left(i\right)\right)}{RMSE\left(I,\hat{I}\left(i\right)\right)}\right)$
***E*_1_***_p_*	111.378	-
***E*_2_***_p_*	3.116	0.972
***E*_3_***_p_*	1.478	0.526
***E*_4_***_p_*	0.034	0.977
***E*_5_***_p_*	0.031	**0.074**
***E*_6_***_p_*	0.010	0.667
***E*_7_***_p_*	0.009	**0.058**

**Table 2.** SA values of endmember set after the second processing step. The values shown in bold italics are those less than the threshold 0.334.

	***E*_1p_**	***E*_2p_**	***E*_3p_**	***E*_4p_**	***E*_6p_**
***E*_1p_**	0.000	0.353	0.901	0.388	0.374
***E*_2p_**		0.000	0.642	0.208	0.031
***E*_3p_**			0.000	0.727	0.626
***E*_4p_**				0.000	0.232
***E*_6p_**					0.000

**Table 3.** RMSEs and rates of change of extracted endmembers. The values shown in bold italics are those of endmembers for which the rate of decrease is less than 0.1.

**Endmember**	**RMSE (**RMSE (*I*,*Î*(*i*))	**Rate of Decrease** $\left(\frac{\left(RMSE\right)\left(I,\hat{I}\left(i-1\right)\right)-RMSE\left(I,\hat{I}\left(i\right)\right)}{RMSE\left(I,\hat{I}\left(i\right)\right)}\right)$
*E*_1_*_p_*	4.959	-
*E*_2_*_p_*	0.200	0.960
*E*_3_*_p_*	0.037	0.815
*E*_4_*_p_*	0.024	0.360
*E*_5_*_p_*	0.020	0.174
*E*_6_*_p_*	**0.019**	**0.007**
*E*_7_*_p_*	0.009	0.524

**Table 4.** SA values of endmember set after the second processing step. The values shown in bold italics are those less than the threshold 0.213.

	***E*_1_***_p_*	***E*_2_***_p_*	***E*_3_***_p_*	***E*_4_***_p_*	***E*_5_***_p_*	***E*_7_***_p_*
***E*_1_***_p_*	0.000	0.218	0.508	0.524	0.621	***0.131***
***E*_2_***_p_*		0.000	0.693	0.672	0.742	0.308
***E*_3_***_p_*			0.000	0.301	0.494	0.444
***E*_4_***_p_*				0.000	0.302	0.478
***E*_5_***_p_*					0.000	0.576
***E*_7_***_p_*						0.000

**Table 5.** RMSE values between ground-truth data and extracted endmembers using the proposed method and the RN-FINDR method on the AISA data.

	**Ground-truth Material**							
	**Grass**	**Tartan Turf**	**Green Fabric**	**Slate**	**White Gravel**	**Soil**	**Average**	**Standard Deviation**
**Proposed method**	0.009	0.006	0.004	0.094	0.029	0.128	0.045	0.048
**RN-FINDR**	0.010	0.018	0.022	0.099	0.030	0.132	0.052	0.046

**Table 6.** RMSEs and rates of change of extracted endmembers. The values shown in bold italics are those of endmembers for which the rate of decrease is less than 0.1.

**Endmember**	**RMSE (RMSE (*I*,*Î*(*i*))**	**Rate of Decrease** $\left(\frac{\left(RMSE\right)\left(I,\hat{I}\left(i-1\right)\right)-RMSE\left(I,\hat{I}\left(i\right)\right)}{RMSE\left(I,\hat{I}\left(i\right)\right)}\right)$
***E*_1_***_p_*	1.612	-
***E*_2_***_p_*	0.518	0.679
***E*_3_***_p_*	0.048	0.908
***E*_4_***_p_*	0.021	0.560
***E*_5_***_p_*	0.020	**0.064**
***E*_6_***_p_*	0.014	0.272
***E*_7_***_p_*	0.013	**0.079**
***E*_8_***_p_*	0.010	0.214
***E*_9_***_p_*	0.010	**0.034**
***E*_10_***_p_*	0.009	0.113

**Table 7.** SA values of endmember set after the second processing step. The values shown in bold italics are those less than the threshold 0.693.

	***E*_1_***_p_*	***E*_2_***_p_*	***E*_3_***_p_*	***E*_4_***_p_*	***E*_6_***_p_*	***E*_8_***_p_*	***E*_10_***_p_*
***E*_1_***_p_*	0.000	0.792	0.699	**0.211**	**0.314**	**0.461**	**0.145**
***E*_2_***_p_*		0.000	0.873	0.862	0.957	0.944	0.784
***E*_3_***_p_*			0.000	0.804	0.972	0.988	**0.613**
***E*_4_***_p_*				0.000	**0.283**	**0.257**	**0.299**
***E*_6_***_p_*					0.000	**0.409**	**0.413**
***E*_8_***_p_*						0.000	**0.537**
***E*_10_***_p_*							0.000

**Table 8.** RMSEs between ground-truth data and extracted endmembers in the CASI data obtained using the proposed method and RN-FINDR.

	**Ground-truth Material**					
	**Water**	**Vegetation**	**Roads**	**Buildings**	**Average**	**Standard Deviation**
**Proposed Method**	0.256	0.126	0.303	0.208	0.223	0.066
**RN-FINDR**	0.289	0.417	0.358	0.180	0.311	0.088
